# Moving Microgrid Hierarchical Control to an SDN-Based Kubernetes Cluster: A Framework for Reliable and Flexible Energy Distribution

**DOI:** 10.3390/s23073395

**Published:** 2023-03-23

**Authors:** Ricardo Pérez, Marco Rivera, Yamisleydi Salgueiro, Carlos R. Baier, Patrick Wheeler

**Affiliations:** 1 Department of Computer Science, Faculty of Engineering, Universidad de Talca, Curicó 3341717, Chile; riperez@utalca.cl; 2 Department of Electrical Engineering, Faculty of Engineering, Universidad de Talca, Curicó 3341717, Chile; 3 Department of Electrical and Electronic Engineering, Faculty of Engineering, University of Nottingham, Nottingham, NG7 2GT, UK; 4 Department of Industrial Engineering, Faculty of Engineering, Universidad de Talca, Curicó 3341717, Chile

**Keywords:** kubernetes, hierarchical control, microgrids, microservices, software defined networking

## Abstract

Software Defined Networking (SDN) is a communication alternative to increase the scalability and resilience of microgrid hierarchical control. The common architecture has a centralized and monolithic topology, where the controller is highly susceptible to latency problems, resiliency, and scalability issues. This paper proposes a novel and intelligent control network to improve the performance of microgrid communications, solving the typical drawback of monolithic SDN controllers. The SDN controller’s functionalities are segregated into microservices groups and distributed through a bare-metal Kubernetes cluster. Results are presented from PLECS hardware in the loop simulation to validate the seamless transition between standard hierarchical control to the SDN networked microgrid. The microservices significantly impact the performance of the SDN controller, decreasing the latency by 10.76% compared with a monolithic architecture. Furthermore, the proposed approach demonstrates a 42.23% decrease in packet loss versus monolithic topologies and a 53.41% reduction in recovery time during failures. Combining Kubernetes with SDN microservices can eliminate the single point of failure in hierarchical control, improve application recovery time, and enhance containerization benefits, including security and portability. This proposal represents a reference framework for future edge computing and intelligent control approaches in networked microgrids.

## 1. Introduction

Microgrid hierarchical control aims to regulate the network frequency and voltage through collaborative work between distributed energy sources. This strategy has changed the impact of distributed generation. However, it has imposed several challenges in power control systems, especially those related to integrating power electronics, telecommunications, fault monitoring, and security issues [1]. The trend of this strategy is to use three levels of hierarchical control to standardize the microgrid’s operation and increase its resilience.

The primary level regulates the network’s frequency and voltage, ensuring power-sharing between the distributed generators (DGs) [2]. The most important approach is the droop control [3], which is based on the droop features of a conventional generator. Droop control ensures stability between frequency or active power (f−P) and voltage or reactive power (V−Q). The secondary level stabilizes the voltage and frequency deviations due to the output impedance decoupling, failures in power-sharing or the presence of circulating currents [4]. At this level, sharing information in real time between the DGs and supporting the stability of the most critical control variables within their nominal values is essential.

The literature provides evidence that integrates the power system with communications architectures in networked microgrids (NMG). For example, refs. [5,6,7,8] proposes a two-level hierarchical control capable of regulating the active and reactive power sharing. However, despite using a low-bandwidth communication system, the topology needed to be more scalable due to the recent development of complex control algorithms. Previously reported research modified the communications architecture and control approach to add new facility units and control algorithms [9]. Furthermore, conventional communications and routing protocols cannot handle multiple topology events and lack sufficient intelligence to make appropriate control decisions [10].

Software Defined Networking (SDN) is a modern approach to networking that allows for controlling and managing network resources and traffic flow through software abstractions [11]. SDN provides greater flexibility and scalability by decoupling the control and data planes and enabling network administrators to configure and manage the topology behavior. This leads to faster deployment times, improved network visibility, and easier troubleshooting [12]. Additionally, it provides new opportunities for network automation, real-time analytics, and increased security through granular policy enforcement. SDN also promotes an open and interoperable network environment, enabling organizations to utilize various vendor offerings and technology advancements.

The main limitation of SDN technology is the use of a centralized control plane and the inconveniences associated with a single point of failure of this critical node. The centralized control plane can potentially lead to network downtime if not properly managed. In [13,14,15], the authors propose a set of systems that aim to solve the drawbacks of conventional SDN technology. However, using a reliable communications strategy is still necessary to improve the heterogeneous communication between different DGs according to the restrictions defined by the architecture and control algorithms. On the other hand, the complexity associated with multiple programming APIs within a monolithic controller requires a high level of technical expertise and represents a significant challenge to network operations. Despite these limitations, different researchers and organizations are allocating resources toward modernizing their power systems and communication networks.

One promising approach is to distribute the functions of the SDN controller into multiple microservices [16,17]. The goal is to implement these functions as distributed units with a replication factor for redundancy. By doing so, the workload and resources of the SDN controller are spread among different worker nodes. In the event of a failure, the data remains accessible through the microservice controller without requiring manual intervention.

There is a wide variety of SDN controllers developed using different programming languages [18,19], among which Ryu [20], Opendaylight [21], and ONOS [22] being the most significant due to their capabilities, ease of deployment, and reliability. A study in [17] explored the development of a microservices-based system for the Ryu controller within the OpenStack virtualized network infrastructure [23]. However, despite dividing the main functionalities of the Ryu controller into Docker containers, these microservices cannot automatically scale in case of failure. In the case of a worker node failure, there is no automatic method to restore the functions assigned to that element, impacting the communication system and the microgrid’s performance. Although this approach offers a fresh perspective on deploying SDN controllers, it must be noted that Ryu was not originally designed to support microservices [24]. The previous drawback requires careful testing of the architecture, services, and communication interfaces before implementation in real-world environments.

μONOS is an SDN controller that uses microservices in large-scale communication networks [25]. The ONOS Project presents μONOS as a solution for disaggregating the functionalities of the SDN controller into microservices. This project aims to enhance the versatility of the SDN architecture by targeting cloud computing platforms, data centers, and bare-metal deployments. A bare-metal cluster removes the hypervisor overhead and puts the Kubernetes installation directly on the host server’s operating system.

An orchestrator that automates network management, load balancing, and new instances provides an intelligent topology that uses microgrid resources efficiently. Combining SDN with Kubernetes provides an intelligent system that analyzes traffic and application requirements in real-time to adjust network configuration and application deployment. Recent literature [26,27] suggests that although there are differences between this proposal and artificial intelligence (AI), the communication alternative can also be classified as intelligent because of SDN’s programmability and the system’s capacity to optimize network performance. Kubernetes manages containerized applications’ orchestration and deployment, while SDN manages the network infrastructure supporting those applications. Together, they can dynamically respond to changes in traffic, adjust network policies, and optimize network performance. For instance, when traffic surges, SDN can automatically increase network resources by adding nodes or expanding bandwidth. Likewise, if an application needs to be migrated, SDN can adapt the network routing to reduce recovery time. Compared to traditional networking, which can be time-consuming and error-prone, our proposal offers automated configuration and management that is not restricted to specific conditions [28].

According to literature [29,30], the restrictions in the communications systems imposed by the penetration of more distributed generation sources force the use of distributed, autonomous and efficient communications strategies to control the power system efficiently. Microgrids need bare-metal Kubernetes to ensure high reliability, low latency, and optimal resource utilization [31], which are critical requirements for a microgrid’s efficient and safe operation. Standby servers may provide low reliability and performance than a Kubernetes-based infrastructure, especially in dynamic and changing load conditions. There are several reasons to propose bare-metal Kubernetes for microgrids:

- Reliability [32]: Bare-metal Kubernetes provides a highly reliable infrastructure for microgrids by distributing the workloads across multiple nodes and ensuring the high availability of resources.
- Computational cost [33]: Low costs because virtualization software is no longer necessary. Cluster automation and microservices deployment are straightforward because there is no hypervisor.
- Low latency [34]: Microgrids require low latency and high-speed communication between the devices to ensure safe and efficient operations. Bare-metal Kubernetes provides low-latency network connectivity and efficient data communication.
- Optimal resource utilization [33,35]: Microgrids require optimal resource utilization to ensure energy efficiency and reduce operational costs. Bare-metal Kubernetes provides efficient resource allocation and utilization, which can help optimize energy consumption and reduce costs.
- Scalability [36]: Network configuration is more straightforward on the bare-metal cluster and troubleshooting. Microgrids require the ability to scale up or down depending on the demand. Bare-metal Kubernetes provides automatic scaling and load balancing, which can help ensure optimal performance under varying load conditions.

Despite the previous comments and the benefits of Kubernetes, there is no evidence of SDN microservices being used to solve the disadvantages of microgrid hierarchical control. Decoupling the applications from the monolithic controller into a series of sub-functions enables the deployment of a highly flexible SDN architecture. The most critical impact of this research is the ability to coordinate different applications as microservices and provide the guidelines for programming APIs in microgrid hierarchical control.

This paper proposes a novel hierarchical control architecture based on microservices to address the limitations of SDN controllers in networked microgrids. Implementing a set of controllers as a distributed system in the Kubernetes bare-metal cluster increases the redundancy and resilience of the communication system. The load is distributed among various devices based on communication and power system restrictions. Rather than using a monolithic controller, the controller functions are divided into a group of microservices. The key contributions of this research are presented as follows.

- A new architecture, based on microservices, as a solution to the centralized SDN controller problem regarding load balancing, scalability, and low latency. The proposed methods improve the global resilience of the system and allow the integration of SDN controllers as pod services in distributed Kubernetes platforms. The proposed approach allows the deployment of bare-metal Kubernetes cluster parameters and can be applied to multiple configurations of AC/DC microgrids.
- A new SDN communication architecture has been developed for hardware-in-the-loop platforms connected to Raspberry pi, serving as both a Kubernetes worker and an OpenFlow communication device. Furthermore, this paper analyzes the most significant drawbacks of the SDN control plane in networked microgrids.
- Provides a proof of concept to apply μONOS for segregating and orchestrating services in bare-metal Kubernetes cluster. The proposed method decreases the data flow traffic through the SDN infrastructure, setting the most appropriate route between the DGs. The distributed communication system is capable of managing real-time energy data.

This implementation can be replicated and modified through our project’s GitHub repository. Furthermore, it designs a monitoring tool integration that allows visualization of logs and measures metrics to carry out a complete analysis of the networked microgrid.

The rest of this paper is organized as follows. Section 2 reviews the main limitations of SDN controllers according to physical architecture, interfaces, reliability, and scalability. Section 3 describes the development of hierarchical control of microgrids and the control method applied. Section 4 provides the microservices benefits of SDN controller functionalities disaggregation and our methodology. The implementation of the SDN controller as a group of microservices is presented in detail in Section 5. The results and discussion of the significance of our proposal are given in Section 6, according to different metrics. On the other hand, Section 7 presents different communication failures and compares the performance of the communication systems. Section 8 concludes the work and provides an overview of future research topics.

## 2. Main Disadvantage of an SDN Controller

This section includes the fundamental disadvantages of SDN controllers, comparing monolithic and centralized architectures versus microservices-based architectures. The contrasted elements allow determining the most relevant metrics that mark the performance of the SDN protocol. The scalability of the communications system proposed by SDN integrated with Kubernetes allows the deployment of an intelligent DG architecture at a reduced cost and increases the overall security through SDN controller functions.

Although Software Defined Networking has several benefits, it has certain limitations that must be considered. Additionally, the security elements of SDN solutions are still a concern, as the centralized control plane can be a target for cyberattacks. The most significant drawbacks of SDN technology are summarized below.

### 2.1. Centralized Controller

The most widely used architecture for microgrid control uses a centralized SDN controller as an intelligent element within the topology. However, critical aspects such as latency, network convergence (less than 100 ms), reliability (close to 99%), and packet losses must be managed carefully [37,38]. Achieving these properties is difficult in a centralized control scheme, especially for a large topology, due to communication devices’ propagation latency and processing time. In this way, centralized SDN controllers have significant challenges, especially regarding scalability and reliability. Consequently, the controller node becomes a key target for cyberattacks. If the information stored in the controller is compromised, there is no way to recover it, resulting in a negative impact on the network and economic losses. A possible alternative is to use distributed SDN controllers as stated in [39,40].

### 2.2. Monolithic Controller

Multiple SDN implementations adopt a centralized controller that relies on a monolithic control plane architecture. All the possible functionalities are included in an extensive control program, which needs to be flexible for changing topology conditions [17]. Controllers often use a set of services from a pool, which can result in some services being unused or restricted by the controller. This feature forces the replication of the entire control implementation in the distributed architecture, which limits controller portability. As a result, developers must carefully prepare the functionalities and ensure integration between the modules. This behavior poses a significant challenge for users who need to implement new services and require fast controller deployment.

Monolithic and microservice architectures are two approaches to building Software-Defined Networking (SDN) solutions. Table 1 compares monolithic centralized SDN controller with microservices controller. A monolithic SDN is simple to implement and deploy but has scalability, flexibility, and fault tolerance limitations. In contrast, microservice SDN is designed to decompose the control plane into smaller, independent services that communicate with each other through APIs. Each service is responsible for a specific control function and can be developed, deployed, and scaled independently.

### 2.3. Variability in Programming Interfaces

Some of the most popular SDN controllers (such as OpendayLight, Ryu, or ONOS [18,41]) use REST API interfaces as a communication mechanism. The RESTful API is a device communication interface to secure information exchange over the hypertext transfer protocol. However, each controller exposes its APIs in a different way, forcing users to modify the request syntax or the programming language according to the specific conditions [16]. This lack of standardization turns the applications into systems that depend on a particular SDN controller.

### 2.4. Dependencies between Applications and Controllers

The close relationship between control applications and the controller type presents a challenge that restricts the ability to reuse applications and module configurations. In several situations, modifying the programming language and readjusting critical plugins becomes necessary due to the interdependence between the modules and the controller’s central component.

### 2.5. Lack of Reliability and Scalability of SDN Controller

The principal reason for the lack of reliability and scalability is the high dependency between the SDN controller and the communication events handled by the control plane. Furthermore, a monolithic architecture is complex to scale into a standalone system due to the relationship between the application of the SDN controller and the communication device. Any failure in one component will result in a cascade failure of the entire system.

## 3. Hierarchical Control Approach

**Figure 1:**
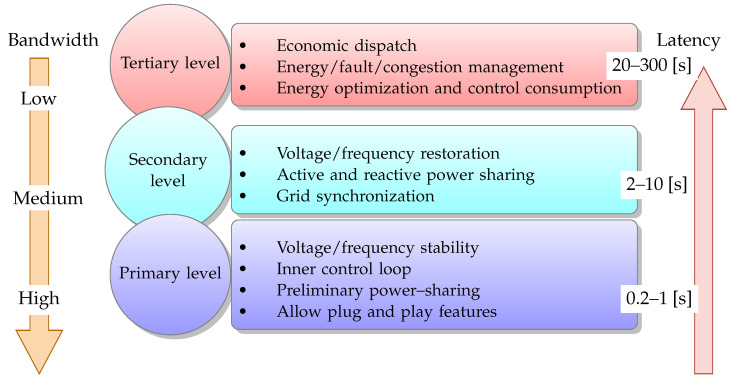
Hierarchical control levels and communication restrictions for networked microgrids.

Hierarchical control is a practical approach to managing power sharing in a Microgrid. The primary control level determines the amount of power to be generated by each DG based on factors such as power demand, availability of energy, and system constraints, as Figure 1 shows. The secondary control level manages the power-sharing between the Microgrid and the utility grid, ensuring that the Microgrid operates within acceptable limits. System optimization and long-term planning are carried out at the tertiary control level to ensure optimal power sharing among the energy sources, energy storage systems, and loads.

**Figure 2:**
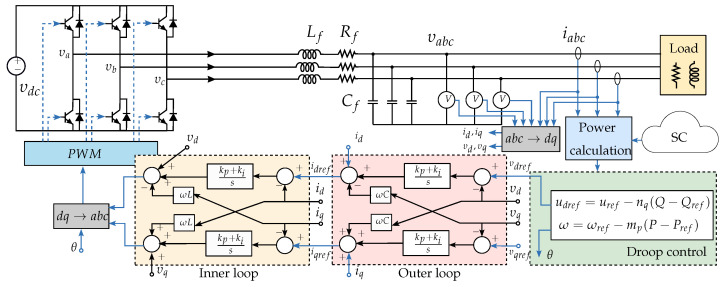
General structure and hierarchical control of one VSI. The acronym SC represents secondary control.

Power sharing between DGs is usually carried out by parallel inverters connected to a common AC bus with multiple loads [42]. The proposed controller in this paper considers the microgrid as three Voltage Source Inverters (VSI) feeding an RL load at the point of common coupling. An RL load was chosen to study the microgrid’s active and reactive power sharing. Each DG represents a power source implemented in The Simulation Platform for Power Electronic Systems (PLECS) [43,44]. Droop control in parallel inverters is a widely used strategy to regulate MG power sharing. The main goal of primary droop control is to set a proportional load sharing among DGs, based on the well-known (P-Q) droop method [5]. Each inverter has an external droop control loop to improve performance and provide a decentralized control method. Figure 2 shows the strategy applied to regulate frequency and voltage for one VSI. The details of hierarchical control can be found in Appendix A and Appendix B.

**Figure 3:**
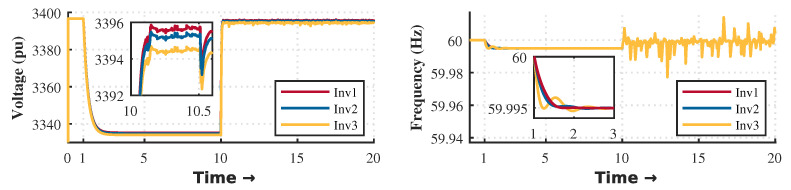
Voltage end frequency regulation with SDN microservice controller of this proposal.

**Figure 4:**
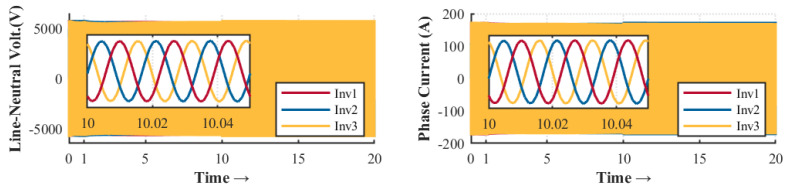
Line to the neutral voltage and phase current for SDN microservice proposal.

The control strategy is evaluated according to different events within the microgrid. The droop control is activated during the first second until it reaches the steady state condition. As shown in Figure 3 and Figure 4, there is a deviation in voltage and frequency that needs to be solved by the secondary control. For that reason, it is necessary to perform secondary control to reach the stability of the MG. The secondary control is activated after 10 seconds and remains until the end of the simulation.

Research studies have used a hierarchical control approach with an SDN-based communication architecture to enhance overall system intelligence [13,37,45]. However, while this approach addresses some aspects of power sharing in networked microgrids, the communication system remains a critical factor that impacts the power system and hinders overall recovery. The monolithic architecture of the SDN controller, the integration of new control functionalities, and the excessive workload can be improved through a microservices-based architecture and automatic orchestration.

## 4. Disaggregating Functionalities and Migrating SDN as Microservices

**Figure 5:**
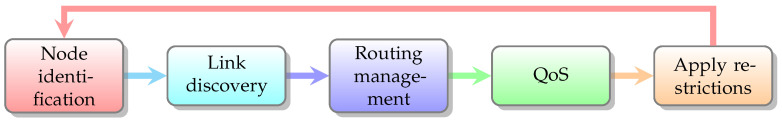
Main functionalities of the SDN controller.

SDN controllers implement different modules to handle the most relevant aspect of the communication devices. These modules afford key functionalities, such as selecting the best route for message forwarding, monitoring topology changes, and enhancing security [46,47], as outlined in Figure 5. The process starts with discovering and managing the nodes, finding the active communication links, and updating functions accordingly. Next, optimal packet flow and routing management are established by creating new flows and forwarding packets. The controller must activate the functions to monitor the traffic and guarantee the quality of service (QoS) according to predefined metrics. Finally, several options are applied to ensure network management and security, avoid latency issues, and improve restrictions imposed by the power system.

Previous functionalities are the components that support segmentation in microservices.

### Components and Interfaces as Microservices

**Figure 6:**
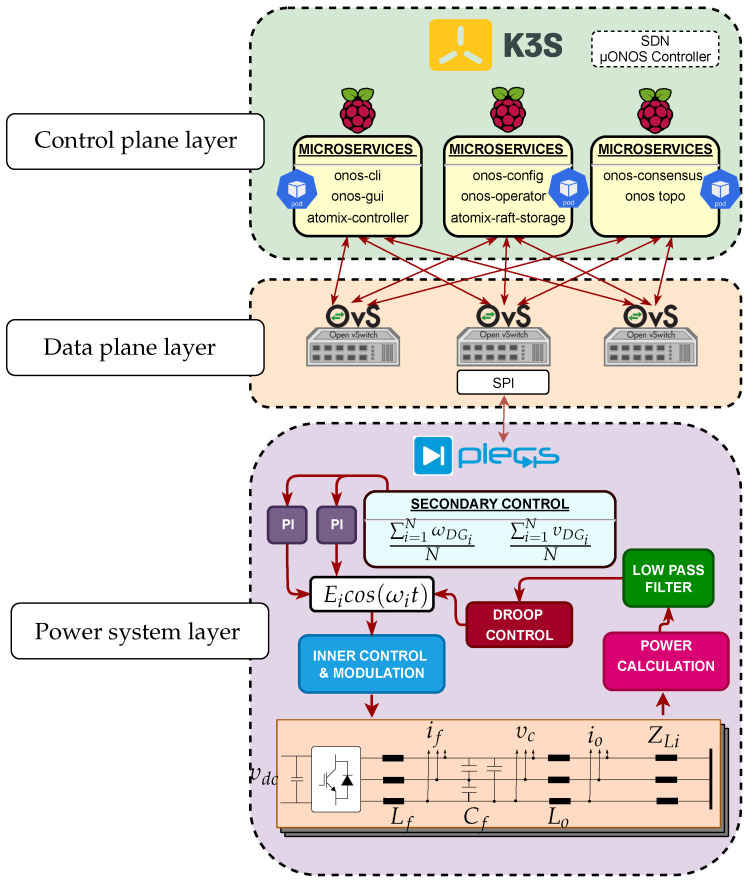
Communication framework for microservices implementation.

In this proposal, the SDN controller deploys a set of microservices in several Docker containers, as shown in Figure 6. The upper layer corresponds to the SDN controller, the middle layer represents the communication topology, and the bottom layer is the electrical system. The container’s functionality includes traffic routing, topology management, and event handling. The ability to segregate the controller functions as microservices allows a distributed system to scale according to topology demand. If the number of containers increases, a platform for orchestrating multiple pods is required.

Figure 6 presents the proposed topology and considers the aggregation of microservices deployed in three Raspberry pi 4 (with ARM architecture). Given the increased computational capacity of the latest versions of Raspberry and its lower cost compared to other computing devices, this platform is selected to operate as a worker in the Kubernetes cluster and an OpenFlow communication device. OpenFlow is a communication protocol SDN uses to standardize the interfaces between the control and data planes [48]. This research uses OpenFlow because it is a standard supported by multiple communication devices, adding great flexibility and programmability to the network. The flexibility of OpenFlow minimizes the effort and resources necessary to manage complex networks, including those in microgrid hierarchical control.

The Kubernetes cluster is deployed in K3s [49] to obtain the SDN controller’s global functionality. K3s is a lightweight Kubernetes distribution built for IoT and Edge computing. The three Raspberrys are physically independent and configured as a high-availability cluster to improve the controller operation and provide good resilience through microservices. The synchronization of each container and the global administration of the SDN controller is orchestrated by Rancher [50]. Rancher is a Kubernetes-based orchestration software integrating container monitoring and management tools through a simple graphical interface.

The northbound interface (NB) allows the interaction between the SDN controller and external applications. On the other hand, the southbound interface (SB) standardizes the operation of the communication protocols, as in the case of OpenFlow [51]. REST APIs are communication channels that use the HTTP protocol to carry out operations such as GET, POST, or DELETE on data. It has high scalability, good performance, and the ability to decouple its functions, making it an ideal candidate for developing microservices.

## 5. Implementation of *μONOS* SDN Controller

Microgrids are changing into more complex and extensive networks in which applications, service virtualization, and edge computing are highly related to control strategies [29,52]. Micro ONOS (*μONOS*) is a new version of the SDN controller, developed by the Open Network Foundation (ONF [53]), which uses microservices to deploy a scalable infrastructure with excellent throughput and low latency. It is based on Docker containers deployed in a cloud-based infrastructure or local data centers. Unlike monolithic controllers that integrate multiple APIs, it comprises a few interfaces such as Google Remote Procedure Call (gRPC), gRPC Network Management Interface (gNMI) and P4Runtime [25].

The μONOS infrastructure, network functions and monitoring services only support Kubernetes applications through Helmcharts implementation [54]. To install, manage and delete the device configurations and their performance, μONOS configures the gNMI as an open-source data management protocol for network devices. The functionalities provided by gNMI can be modeled using YANG (Yet Another Next Generation data modeling language) [25]. The network manager interacts with the SDN controller through the gRPC interface, accessing both the onos-cli and onos-gui services for network management, network modifications and reverting changes. According to ONF [53], the onos-config service offers a gNMI endpoint for various functions, including reading states, configuring settings, and subscribing to specific features. This interface can also prevent invalid values and enable the operational state.

**Figure 7:**
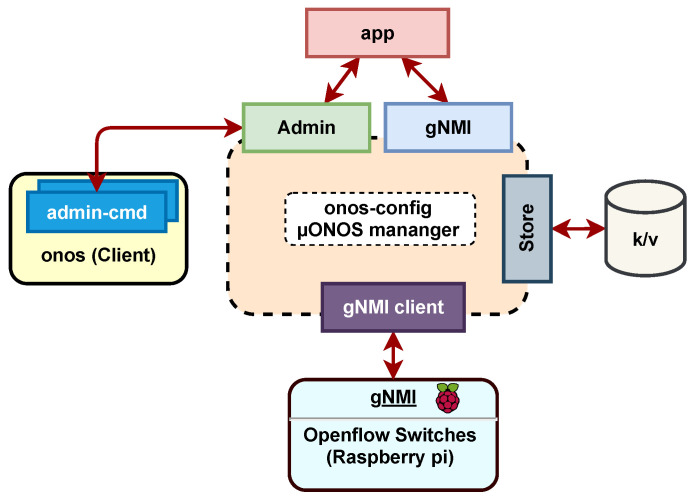
High-level design of the proposed μONOS controller (modified from [25]).

The μONOS identification component facilitates the controller’s management from external applications, as depicted in the top layer of Figure 7. A proposed middleware enables information exchange between external applications (power-sharing information from networked MG) and the SDN control plane. As shown in Figure 7, the core of the SDN controller is separated from the event handler within the middleware, allowing communication between the REST API and the controller microservices. This service, named onos-config, is responsible for linking the module’s functionality to the specific microservices that are executed in a distributed manner.

The essential microservice of Figure 7 is the block onos-config, as it manages the configuration of devices through gNMI interfaces and registers all events to send them to the Atomix driver. The Atomix driver implements an API to scale the μONOS Kubernetes resources. This property of scaling resources adds greater redundancy to the controller. It provides a system with distributed additional resources and is responsible for maintaining and managing the µONOS service. Atomix controller details can be found in the GitHub repo of the Open Networking Foundation [55].

### 5.1. Functionalities of onos-config Module

The onos-config module strives to handle network and device alterations by invoking the NetworkChange and DeviceChange services, respectively. These services keep records of all change logs and pass them to the Atomix drivers via the gRPC interface.

For connecting devices through the southbound interface, onos-config only supports it through gNMI. Furthermore, the YANG models set the configuration and topology architecture into onos-config service. The deployment of this module requires a Kubernetes cluster capable of running Helmcharts as detailed in its deployment page [56]. The interaction between onos-config and onos-cli allows the user to define rules or route paths through the gNMI interface. To execute it, access the onos-cli pod and run the plugins from there (you can view the list of plugins by running onos-config get plugins).

**Figure 8:**
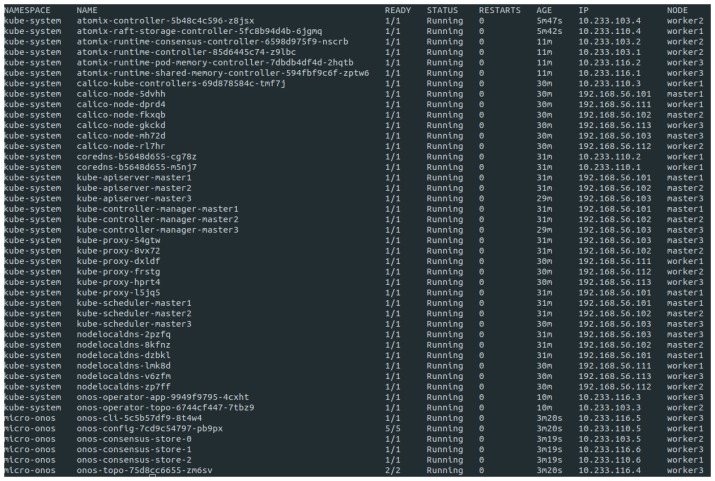
Number of pods of the SDN controller distributed in the worker’s nodes.

Figure 8 shows the deployment of the pods during the execution of the μONOS in the topology. Five pods of the onos-config service are deployed, and six pods of Atomix increase the availability and distribute the services across the nodes. All steps to deploy this proposal are available in the GitHub repository [57].

### 5.2. Network Interface Cluster Implementation

**Figure 9:**
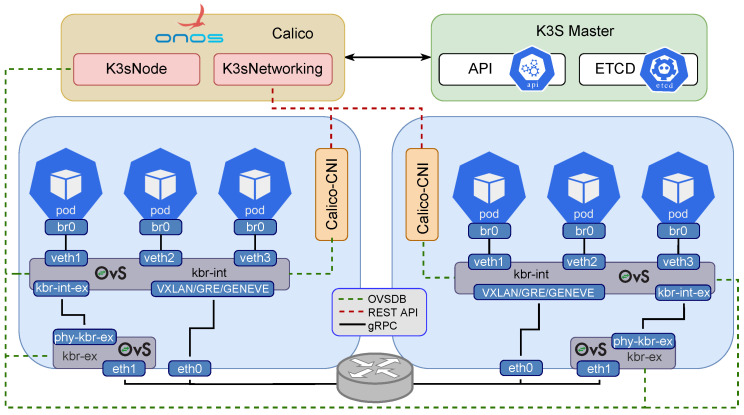
Kubernetes management and overlay tunneling networking with Calico-CNI.

This paper implements two network interfaces for each Raspberry, as shown in Figure 9. The eth0 is the default ethernet interface of the Raspberry pi 4. It serves as the Linux bridge and is used for configuring Kubernetes clusters, exchanging pods control information, accessing Rancher and the load balancer, and connecting to the Internet gateway. It means that eth0 acts as an overlay interface for external communications. On the other hand, eth1 is used by the OpenFlow protocol to communicate between SDN service pods. Conversely, the eth1 interface, connected via a USB to Ethernet adapter, enables the substitution of the veth interfaces of the pods with kbr-int-ex. Each pod in the cluster will use the kbr interface instead of its veth to avoid routing and forwarding issues.

Calico CNI (Kubernetes Container Network Interface (CNI)) deploys a Daemonset on each node, ensuring communication between gRPC and the μONOS controller. In other words, this plugin provides networking for the containers and pods within the Kubernetes cluster. The cluster formed by the Raspberry nodes can operate as conventional Ethernet devices (via TCP and Unix domain socket) or as an OpenFlow switch to handle SDN traffic.

### 5.3. Create the Kubernetes Cluster on Raspberry

K3s distribution [49] allows deploying of the bare-metal cluster and is an excellent choice for IoT devices, particularly Raspberry pi. From Rancher’s official documentation [58], a high-availability cluster with an embedded database is implemented. However, using a single load balancer as implemented in the default configuration brings back the drawbacks of the centralized system. Integrating the K3s cluster with Keepalived and HAproxy as described in [59], solves the previous disadvantages in a distributed way. To set up an HA Kubernetes cluster using Keepalived and HAproxy, you need to install and configure Keepalived and HAproxy on each node in the cluster. Keepalived is used to manage the virtual IP address that clients use to access the Kubernetes API. In contrast, HAproxy is used to load balance incoming traffic across the Kubernetes API server nodes. This alternative is superior to the external database implementation because the storage is distributed in each etcd node’s services [58], increasing the system’s availability and removing the single point of failure through distributed storage.

Ansible automates node creation, storage configuration, network interfaces, services, and deployment processes. This tool allows (in a simple way) the cluster to be deployed through the execution of a series of scripts developed in Ansible. All the manifest and Ansible inventory files are in the shared GitHub repo.

### 5.4. Connection to PLECS RT Box

Possible communication alternatives between Raspberry and PLECS are SPI, I2C, and CAN protocols. However, the PLECS RT Box only has two SPI communication modules (SPI1 and SPI2), leaving one of our worker nodes unconnected. In [60], the average data rates of the three technologies are compared. According to the PLECS manual, SPI has the best data rate, followed by the CAN bus and I2C. We decided to connect the third node of the cluster to the same SPI port of node 2’s PLECS server for these reasons. Although this is not the best communication alternative, as the frequency and voltage values on each Raspberry are averaged, this will not affect the overall performance of the MG. For more complex microgrid implementations, it is recommended to use the SFP transceiver modules, available from PLECS, to achieve speeds of up to 10Gbps.

**Figure 10:**
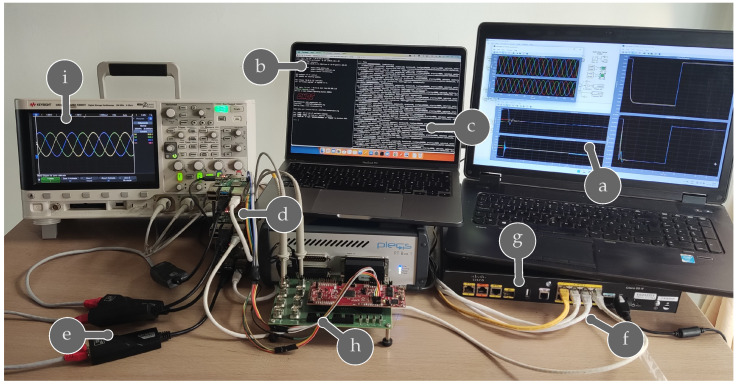
Experimental setup for Kubernetes cluster with Raspberry pi: (a) PLECS output, (b) ONOS command line interface, (c) SDN flows during communication, (d) Raspberry pi Kubernetes cluster, (e) USB network adapter, (f) overlay interface, (g) Cisco router and 52switch, (h) SPI connection, (i) MG output current.

Each Raspberry will serve as an OpenFlow communication device that ensures the exchange of secondary control information. Figure 10 shows the practical implementation of this proposal. There are three Raspberrys connected through Ethernet and USB. The first connection allows external access, and the second provides SDN functionalities. Additionally, the PLECS platform is wired to the cluster through the SPI bus of the Launchpad F28069M.

According to the price of this proposal, Table 2 shows that it is possible to create a bare-metal cluster with less than 1300 USD. The Cisco router can be replaced by other cheaper alternatives, such as the ZodiacFX [61].

### 5.5. Monitoring Platform

Different factors, such as hardware resources, load, or communication architecture, limit the number of pods executed on each device. However, with Rancher, these pods can be scaled automatically without compromising the cluster’s overall structure. To configure automatic scaling in Rancher is necessary to select the deployment. From there, the “Scaling” tab specifies the minimum, maximum, and desired number of replicas for the deployment and also sets the scaling policy based on CPU or memory usage. This proposal shows the number of replicas for each service in Figure 8, with the boundary for scaling new pods set at 80% of CPU usage. It’s important to note that automatic scaling requires a monitoring and metrics system to track your deployment’s resource utilization. Rancher integrates with Prometheus and Grafana to trigger alerts according to the threshold values configured. Finally, the Grafana graphical user interface allows monitoring the deployment’s status and the number of replicas scaled.

**Figure 11:**
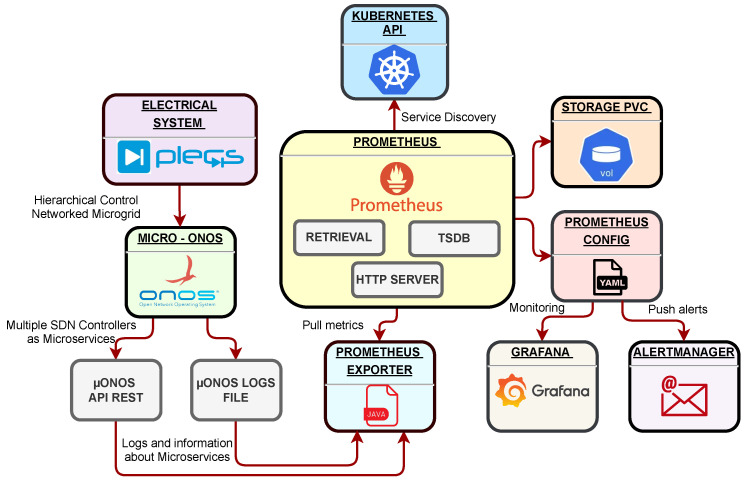
Proposed architecture for monitoring and integration of the electrical system, SDN topology, and Cloud Computing environment.

The solution to monitoring the communication devices’ status and the tools’ interaction is presented in Figure 11. Our proposal uses a Rancher Helmchart to develop a Java application (Prometheus Exporter) to obtain information about network metrics. Furthermore, it exports the data to Prometheus, which is responsible for monitoring events and triggering alerts according to standard conditions.

Prometheus sends the information to the local storage to collect operating system metrics. We use the μONOS interface to obtain information and network metrics. Different notifications can be triggered through the Prometheus configuration file to perform fast reactions without downtime.

**Figure 12:**
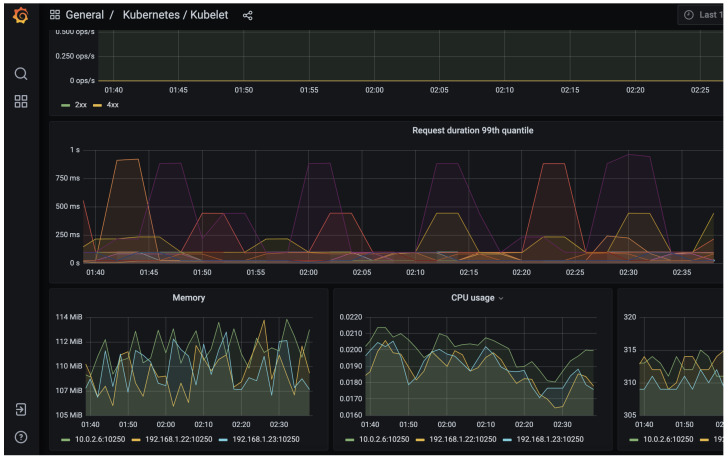
Types of events in Grafana tool. Packets out to PLECS with destination microservices in K3S cluster. Grafana registers data.

Finally, Grafana allows importing a series of dashboards with information on the DG flows. This tool obtains the knowledge of the packet flows that pass through the USB ethernet adapter to the SDN controller. Figure 12 demonstrate the correct integration of Prometheus and Grafana within the Kubernetes cluster. Using Rancher in this proposal simplifies the deployment and configuration of the services.

An essential element to consider when implementing a high-availability Kubernetes cluster on Raspberry is the performance of the computational resources. As the cluster scales, the computational resources become more limited. Increasing the number of pods in Kubernetes can be done to minimize the impact on service performance as follows.

- Ensure that the nodes in your cluster have enough resources (CPU, memory, storage) to support the increased number of pods. The notification system and the alerts configured in Grafana allow the monitoring of resource usage and global capacity.
- Using horizontal pod autoscaling (HPA) automatically adjusts the number of pods based on resource usage and demand. HPA can be configured based on CPU usage, memory usage, or custom metrics.
- To prevent resource contention and performance issues, pod anti-affinity rules ensure that pods are not placed on the same node. This method avoids the scheduling of pods on the same node.
- Optimize pod resource requests and limits to function correctly.
- Use pod disruption budgets (PDB) to ensure that a minimum number of pods are available during node maintenance or failures. By setting a PDB, you can guarantee that the service is unaffected by removing pods from the cluster.

Monitoring the service’s performance following the previous points and adjusting the settings as necessary to optimize resource utilization and performance is crucial.

## 6. Experimental Scenarios and Results

**Figure 13:**
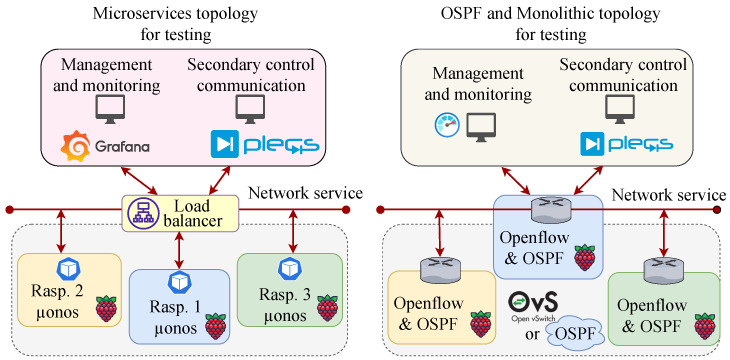
Proposed architecture for testing the metric performance.

To analyze the results of this proposal, a series of critical elements were tested, which imposed restrictions on the communications and power systems. Figure 13 present the topology used to test the microservice (left side) and monolithic/OSPF (to the right side). OSPF and monolithic only differ in the setup of Raspberry configuration. Each Raspberry was configured as a router for OSPF, while the monolithic was configured as an OpenFlow switch. Table 3 shows the main parameter settings of the experiments. The first scenario tested is latency, introduced by decoupling the controller functionalities into distributed microservices.

### 6.1. Latency

**Figure 14:**
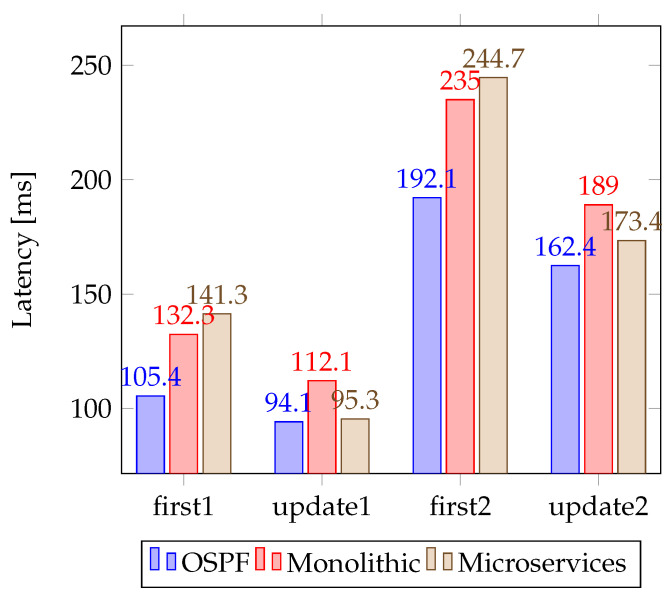
Comparison of latency between OSPF, SDN with a monolithic controller and SDN with the microservice controller. “First” columns represent the latency for the first message and “update” columns represent the latency when the route is established.

The evaluation tests of this proposal consider the measurement of two types of latency. The first column in Figure 14 considers the response time when sending the first message from the MG architecture. This latency gives us a measure of the initial cost of the distributed controller as microservices and the time it takes to obtain a valid route. Once the entry in the flow table is updated, the rest of the packets do not need to go through the SDN controller, so the rest of the flows are expected to have lower latency. The columns update1 and update2 in Figure 14 represent this type of test.

The second experiment considers the average latency for the rest of the packets using OSPF routing and SDN technology. As shown in Figure 14, the performance of the OSPF-based strategy is slightly better. This is due to the additional processing overhead added by the distribution of microservices in three different Raspberry nodes to the processing time of the applications. The complexity of the network topology, as determined by the number of OpenFlow switches, notably impacts the number of controller connections and the processing capacity of Docker containers. The connections between two DGs are denoted by first1 and update1, while the connections between DG1 and the SDN controller are represented by first2 and update2. In OSPF, the connection is established between the two nearest DGs. As can be seen, the highest latency is originated when the first flow is sent between two nodes. Power data (frequency and voltage) shared by hierarchical control in PLECS are shipped using the SPI and CAN protocol, and the results are summarized as average round trip time.

However, even though OSPF and SDN with centralized control have slightly higher performance, the need to use a load balancer is evident, especially in the topology with a monolithic SDN controller and multiple DGs. Furthermore, when the traffic increases, its performance starts to degrade. For the OSPF routing strategy, it can be seen that the sending of the first packet of the topology is slightly higher than its competitors. This is mainly due to the neighbor discovery algorithm and the SPF algorithm needing to know the entire topology for proper performance. To avoid a single point of failure, the proposal of this paper incorporates a distributed load balancer, following the recommendation in [59].

**Figure 15:**
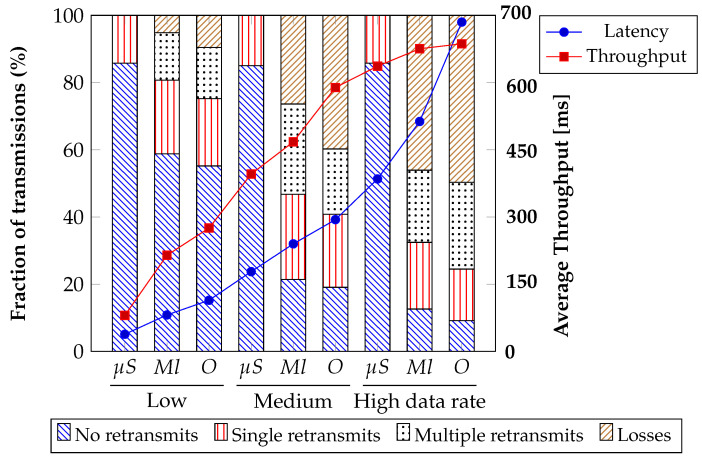
The trade-off between latency and throughput varies across different test scenarios. Columns μS refers to this proposal, Ml means Monilithics architecture and OSPF is *O*.

The researchers performed three communication tests to evaluate the response of the proposed alternatives as a solution to the hierarchical control problem in electrical microgrids. Figure 15 illustrates the percentages of packets successfully delivered on the first transmission, those requiring single retransmission, those requiring multiple retransmissions, and those experiencing packet loss for each scenario. Furthermore, Figure 15 presents a trade-off between monolithic and microservices architectures regarding latency and throughput. Ten simulations were conducted for each of the three tests, and the resulting averages were analyzed to investigate the aforementioned relationship across different data rates. Specifically, low data rates (0<Throughput≤299 Mbps), medium data rates (300<Throughput≤599 Mbps), and high data rates (Throughput≤600 Mbps) were covered in the analysis. The message throughput is modified from the Plecs simulation, increasing the sample period of secondary control. This graph shows the average loss rate for the centralized communication strategies is similar (with a difference of about 5%). In comparison, the distributed systems improve these results by 25 to 60% for the tests performed. The average latency was 287 ms for the global test. The latency increases as the throughput increase due to the closeness to the speed limit supported by the Raspberries.

The findings reveal that monolithic architectures can offer lower latency since all the system components are closely integrated and communicate directly, reducing network communication overhead. However, this close coupling can also limit the system’s horizontal scalability and ability to handle high throughput requirements. On the other hand, microservices architectures can provide higher throughput as the system can be scaled horizontally by adding more instances of individual services as needed. However, the added network communication between services can increase latency and reduce the system’s response time. Typically, the Raspberry Pi 4 (ARM64) can achieve consistent transfer rates of 600–700 Mbps with appropriate network configuration and optimization. Therefore, the choice between monolithic and microservices architectures depends on the system’s requirements. A monolithic architecture may be the better choice if low latency is critical and high throughput requirements are manageable with a tightly-coupled system. A microservices architecture may be more appropriate if high throughput is critical and latency can be tolerated with loosely-coupled services. Our proposal handles the surge in demand by increasing the transmitted packets. In contrast, the monolithic architecture and OSPF demonstrate reduced resilience, scalability, and multiple retransmissions.

In this way, it is evident that the three strategies have an acceptable behavior according to Table 4. However, OSPF and SDN microservices are two different approaches to network management, and they have distinct characteristics and features. The results of Table 4 can be summarized as follows.

- Latency: Regarding latency, OSPF is a distributed protocol that relies on exchanging routing information between devices. It’s designed to find the shortest path between two points, which can help to minimize latency. In general, monolithic architectures can offer lower latency since all the system components are closely integrated and communicate directly, reducing network communication overhead. However, this close coupling can also limit the system’s horizontal scalability and ability to handle high throughput requirements. On the other hand, SDN microservices rely on a central controller that manages the network, and the latency can be affected by the communication between the controller and the devices.
- Throughput: OSPF is a protocol that supports link-state routing and can quickly adapt to network topology changes. As a result, it can provide high throughput in a stable network environment. In contrast, SDN microservices can provide higher throughput as the system can be scaled horizontally by adding more instances of individual services as needed.
- Recovery time: OSPF is designed to support fast convergence and can quickly recover from a link or device failure. However, the convergence time can depend on the size and complexity of the network. SDN microservices can also provide fast recovery times, but it depends on the specific implementation and configuration.
- Link failure: OSPF can detect a link failure and reroute traffic along an alternate path, which helps to maintain connectivity. SDN microservices can also detect link failures and potentially provide more granular control over how traffic is rerouted.
- Device failure: In OSPF, if a device fails, the routing tables are recalculated, and the network can continue to operate. In SDN microservices, the central controller can detect a device failure and reconfigure the network accordingly.
- Controller failure: In SDN microservices, the central controller is a single point of failure. If the controller fails, the network may not be able to operate correctly. However, many SDN solutions provide redundancy and failover mechanisms to minimize the impact of controller failure.

Overall, OSPF and SDN microservices have different strengths and weaknesses, and the choice of which one to use depends on the specific network requirements and goals. All of these values are within the ranges defined by the IEEE 61850 standard [37] for safe microgrid operation. Nevertheless, this microservice implementation has the best portability (by using a Docker container), resiliency (provided by Rancher orchestrator), and scalability results (demonstrated by the recovery time presented in Table 5).

### 6.2. Throughput

Throughput is generally used to determine how well SDN routers and controllers can handle traffic and how efficient they are at this task. This test measures the number of packets sent per second in the case of OSPF, while SDN measures the number of flows installed on the devices. Equation (1) presents a simple way to calculate the throughput.

(1) Throughput=maximum_receiver_bandwidth/round−trip_time

The iperf3 tool obtains the maximum receiver bandwidth, while a simple ping returns the round-trip time. Figure 15 shows the results of this test. As is evident, the results show a better performance in the case of conventional strategies. However, this advantage may be compromised in more extensive networks where the OSPF protocol needs to describe all router neighbors.

## 7. Communication Failure and Recovery Test

Combining Kubernetes with a set of SDN microservices can improve application recovery time by eliminating the single point of failure in hierarchical control. In a traditional network architecture, the control plane and the data plane are tightly coupled, which means that a failure in the control plane can lead to significant disruptions in the network’s operation. This hierarchical control model has a single point of failure, which can be a bottleneck for recovery time.

However, with Kubernetes and SDN microservices, the control plane is decoupled from the data plane, and the control functions are distributed across the network. If a failure occurs in one part of the network, the rest can continue normally, and recovery time can be significantly reduced. Moreover, the combination of Kubernetes and SDN microservices offers a significantly automated and customizable network setting that enables quick and flexible network topology adjustments in response to any modifications in the infrastructure or application. This attribute further reduces the network reconfiguration time, hence enhancing the recovery period, which would otherwise require manual intervention. Combining Kubernetes with SDN microservices can improve application recovery time by eliminating the hierarchical control’s single point of failure and providing a highly automated and programmable network environment. This can help ensure that applications and services are always available and performing optimally, even during network failures.

A communication system failure (closer to the distributed generation sources) is simulated in this scenario. The objective is to verify which strategy has better performance from the point of view of communications without degrading the power-sharing between the local controllers of the MG.

**Figure 16:**
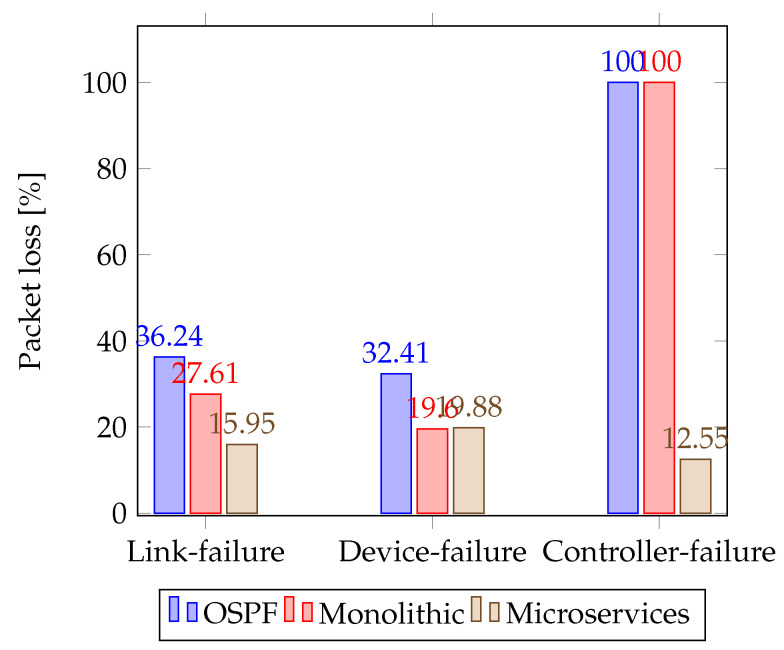
Number of packet loss during convergence time.

The failure is generated, for instance, containing the SDN monolithic, and they are compared with the losses produced in one of the instances that include the microservices. The orchestrator is expected to be able to instantiate a new subsystem instance without degrading the performance of the MG. Kubernetes has been configured to be scaled automatically according to the research shown in [62]. Furthermore, in Figure 16, the packet loss percentage shows that microservices perform well due to distributed services above the nodes.

**Figure 17:**
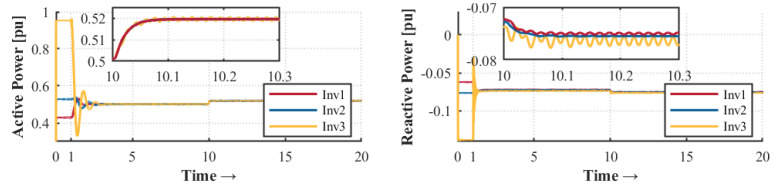
Active and reactive power sharing with SDN distributed controller in this proposal.

**Figure 18:**
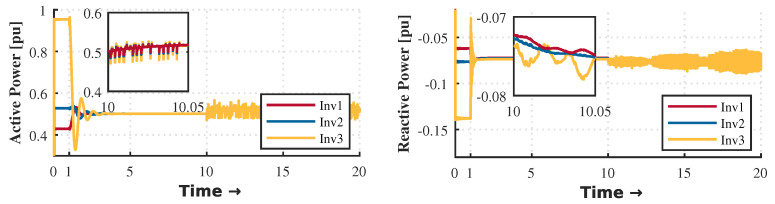
Active and reactive power sharing with SDN monolithic controller.

Figure 17 and Figure 18 show the results obtained during power-sharing with and without hierarchical control, respectively. At one second, the droop control is started to distribute the active and reactive power-sharing. At 10 s, the hierarchical control is enabled to regulate voltage and frequency deviations. Since there is no message loss between controllers (minor glitches only in the average message delay), it proves the system’s robustness. However, the secondary control with a monolithic controller is highly susceptible to small latencies, CPU burden, and propagation delay. In this scenario, one of the OpenFlow switches was removed to determine the effects of the monolithic control strategy. This experiment should be understood as a way to highlight the robustness achieved by the SDN system in its deployment based on microservices.

Our proposal uses a proactive approach to latency testing and a reactive approach to manage topology changes. The architecture will react immediately if a new distributed generation source is added, allowing proper power sharing, as Figure 3 shows. On the other hand, the orchestrator can add intelligence to the topology according to its appropriate programming. For example, our monitoring system architecture can detect latency increasing or node congestion and take the necessary actions to reduce the impact and the consequences. For example, it can scale a more significant number of instances as microservices and thus simultaneously serve more communication requests.

## 8. Conclusions

Monolithic controllers have several drawbacks concerning scalability and reliability. In most cases, these architectures do not meet the requirements of fault tolerance and rapid adaptability, which are imposed by networked microgrids. This proposal’s most significant contribution was the property to dynamically reconfigure control flows based on a microservices architecture and the automatic deployment of microservices instances.

Using a bare-metal Kubernetes cluster on a Raspberry pi and deploying distributed microservices allowed us to improve the reality of a distributed energy control system. Microservices testbeds demonstrated the SDN controllers’ rapid deployment, portability, high availability and resiliency to application failures.

The Kubernetes orchestrator provided good scalability of the communication system, as well as improved the fault tolerance and replication capacity. This is due to the high fault tolerance, capable of managing and distributing the load between microservices. From a comparative perspective, this proposal significantly improves failure recovery time and resilience concerning communications devices.

The API REST microservice topology allowed the splitting of the SDN controller’s core functionalities into small, well-defined functions. In all test case scenarios, reliability showed excellent behavior. Furthermore, the portability of all the nodes in the topology is possible due to the Docker containers. The ability to exchange control information between DGs over an SDN network allows them to regulate the system’s response and reach a steady state more quickly. The results show that to increase the resilience of the network, more sophisticated control strategies and highly available programmable communications networks are required. Finally, the monitoring architecture allows the export of logs in real-time and detects failures through notification software.

## Figures and Tables

**Table 1:** Comparison of monolithic centralized SDN controller vs. microservices controller.

Metric	Monolithic	Microservices
Resiliency	The whole system can be affected by a bug, communication or device failure, or security issues.	Other services are not affected by a failure in a particular microservice.
Deployment	Simple and fast deployment architecture.	Orchestrating the deployment becomes complex due to communication and hardware. restrictions.
Scalability	Redeploying the entire system to manage new changes make it difficult to manage and maintain.	You can scale each element independently without experiencing any downtime.
Compatibility	Adopting new technology languages or frameworks is impossible due to the lack of flexibility.	Multiple integration and standardization.
Security	Communication within a single unit secure data processing.	The use of APIs to communicate different services produces some security threats.
Development	The huge indivisible database makes distributing the team’s efforts impossible.	Each component can be independently operated by a team of developers.

**Table 2:** Total cost of bare-metal cluster.

Item	Quantity	Unit Price in USD
Raspberry pi 4B 8 GB	3	170
SanDisk Micro SD card 32 GB	3	5
Adapter USB to Ethernet	4	10
0.5 m CAT6 Ethernet cables	4	4.5
Router Cisco 891F (not necessary)	1	670
Total cost		1248

**Table 3:** Electrical and control parameters of the MG.

Item	Value
Microgrid parameter	
Rated frequency	60 [Hz]
Rated voltage	$4160\sqrt{2/3}$ [V]
Load power rating RES1	1 [MVA]
Load power rating RES2	500 [kVA]
Load power rating RES3	200 [kVA]
$H_{Line_{1}}$	0.4 [mH]
$H_{Line_{2}}$	0.65 [mH]
$H_{Line_{3}}$	0.9 [mH]
Filter (L,C)	1.8 [mH], [25 µF]
$R_{o}$, $L_{o}$, $C_{o}$	80[Ω], 12.5 [mH], 5 [mF]
Sample time (Ts)	10 [kHz]
Primary control parameters	
P - ω Droop Coeff. $\left(m_{p}\right)$	1 [$\frac{rad}{W\cdot s}$]
Q - *V* Droop Coeff. $\left(n_{q}\right)$	25 [$\frac{V}{Var}$]
Frequency proportional term $k_{p}f$	0.01
Frequency integral term $k_{i}f$	3 s${}^{-1}$
Voltage proportional term $k_{p}\upsilon$	0.01
Voltage integral term $k_{i}\upsilon$	2 s${}^{-1}$
Secondary control parameters	
Frequency proportional term $k_{p}f$	0.001
Frequency integral term $k_{i}f$	4 s${}^{-1}$
Voltage proportional term $k_{p}\upsilon$	0.001
Voltage integral term $k_{i}\upsilon$	6 s${}^{-1}$

**Table 4:** Summary of communication results for test scenarios.

Microservices vs. OSPF	Paired Differences			Improvements
	Mean	Std. Deviation	Std. Error	
Latency of first package	−17.41	13.12	2.39	−29.74%
Overall Latency	−20.66	32.41	6.01	−4.75%
Throughput	14.28	5.24	0.95	−4.15%
Recovery time	214.82	39.95	7.29	53.41%
Packet loss-link failure	19.75	2.06	0.37	55.98%
Packet loss-device failure	11.83	2.30	0.42	38.66%
Packet loss-controller failure	86.00	1.38	0.25	100%
**Microservices vs. OSPF**	**Paired Differences**			**Improvements**
	**Mean**	**Std. Deviation**	**Std. Error**	
Latency of first package	8.46	14.02	2.56	−5.09%
Overall Latency	32.91	38.79	7.20	10.76%
Throughput	−50.47	5.96	1.08	7.05%
Recovery time	257.50	36.23	6.61	36.58%
Packet loss-link failure	13.01	2.21	0.40	42.23%
Packet loss-device failure	−1.13	2.41	0.44	−1.42%
Packet loss-controller failure	85.83	1.38	0.25	100%

**Table 5:** Recovery time of different protocols when a failure occurs.

Communication Protocols	Recovery Time
OSPF	637.8 ms
Monolithic controller	468.5 ms
Microservices controller	297.1 ms

## Data Availability

Not applicable.

## Nomenclature

The following abbreviations are used in this manuscript:

API	Application Programming Interface
SDN	Software-defined networking
DGs	Distributed generators
NMG	Networked microgrids
PLECS	The Simulation Platform for Power Electronic Systems
μ ONOS	Microservices Open Network Operating System
Bare-metal	Physical device designed to run dedicated services
AC/DC	Alternating current/direct current
REST API	Representational state transfer for application programming interface
VSI	Voltage source inverter
RL	Resistive-inductive load
P, Q	Active and reactive power
MG	Microgrid
ω	Frequency
$\omega_{ref}$, $\upsilon_{ref}$	Nominal frequency and voltage
ΔP, ΔQ	Power input error for droop control
$m_{p}$, $n_{q}$	Constant to handle maximum deviation of the microgrid
$P_{ref}$, $Q_{ref}$	Nominal frequency and voltage
$\upsilon_{dref}$	Droop control voltage
$\upsilon_{abc}$	Voltage across the filter
α, β	α, β constant frame
$i_{f}$, $i_{o}$	Filter and output currents
$\delta \omega_{DG_{k}}$	Frequency obtained by secondary control
$\delta \upsilon_{DG_{k}}$	Voltage obtained by secondary control
$k{}_{p_{\omega }}$, $k{}_{i_{\omega }}$	Controller parameters of PI
$\omega_{DG_{k}}$, $\upsilon_{DG_{k}}$	Average frequency and voltage broadcasted by each DG
QoS	Quality of service
K3s	Lightweight Kubernetes
ONF	Open Network Foundation
gRPC	Remote Procedure Calls
gNMI	gRPC Network Management Interface
gNOI	gRPC Network Operations Interface
NB, SB	North bound and south bound interfaces
P4Runtime	Control plane specification for controlling the data plane elements
YANG model	Yet another next-generation data modeling language
CNI	Kubernetes container network interface
CAN	Controller area network protocol
SPI	Serial peripheral interface
I2C	Inter-Integrated Circuit communication protocol
OSPF	Open shortest path first communication protocol

## Appendix A

### Primary Control

The primary control includes outer control loops (for voltage regulation), inner control loops (for current regulation), and droop control loops to share the power between inverters. Grid-forming (comprised of the inner and outer loops) includes a proportional-integral PI controller to regulate the output voltage and frequency of the MG. Furthermore, the frequency and voltage amplitude can be addressed by droop control as shown in the Equations (A1) and (A2), [1]:

(A1) $$\omega =\omega_{ref}-m_{p}\left(P-P_{ref}\right)=\omega_{ref}-m_{p}\Delta P$$

(A2) $$\upsilon_{dref}=\upsilon_{ref}-n_{q}\left(Q-Q_{ref}\right)=\upsilon_{ref}-n_{q}\Delta Q$$

where $\omega_{ref}$ and $\upsilon_{ref}$ are the nominal frequency and voltage, *P* and *Q* are the measured active and reactive power injection, and ΔP and ΔQ are the corresponding power input errors for the droop controller. Coefficients $m_{p}$ and $n_{q}$ regulate maximum deviations allowed in the MG [42,63].

Different lengths of transmission lines make the VSI output impedance different, causing unequal power sharing in droop control. Virtual impedance is an essential concept [64] to fix the output impedance value and decouple the control of active and reactive powers. The virtual impedance can help to keep the voltage within certain limits and is used for applications such as harmonic voltage compensation and improved stability. Refs. [37,65] show the implementation of virtual impedance. However, it is out of the scope of this research.

The voltage across the capacitor $\upsilon_{abc}$ can be calculated using Equation (A3):

(A3) $$\upsilon_{abc}\left(t\right)=\underset{\upsilon_{\alpha }^{*}\left(t\right)}{\underset{︸}{\upsilon_{ref}sin\left(\omega_{ref}t\right)}}+\underset{\upsilon_{\beta }^{*}\left(t\right)}{\underset{︸}{j\upsilon_{ref}cos\left(\omega_{ref}t\right)}}$$

where $\upsilon_{ref}$ and $\omega_{ref}$ are the voltage amplitude and angular frequency $\omega_{ref}=2\pi f_{ref}$ of the reference signal in time *t*. The voltage derivative reference is obtained from Equation (A4):

(A4) $$\frac{d\upsilon_{abc}\left(t\right)}{dt}=\underset{\omega_{ref}\upsilon_{\beta }^{*}\left(t\right)}{\underset{︸}{\omega_{ref}\upsilon_{ref}cos\left(\omega_{ref}t\right)}}+\underset{\omega_{ref}\upsilon_{\alpha }^{*}\left(t\right)}{\underset{︸}{j\omega_{ref}\upsilon_{ref}sin\left(\omega_{ref}t\right)}}$$

To track $\upsilon_{ref}$ it is necessary to expand the last results to α,β frame. The predicted currents $i_{f}$ and measured currents $i_{o}$ are calculated as [64], to predict the capacitor derivative voltage as follows:

(A5) $$\frac{d\upsilon_{abc}\left(t\right)}{dt}=\underset{\frac{d\upsilon_{abc}\alpha \left(t\right)}{dt}}{\underset{︸}{\frac{i_{f\alpha }\left(t\right)-i_{o\alpha }\left(t\right)}{C_{f}}}}+j\underset{\frac{d\upsilon_{abc}\beta \left(t\right)}{dt}}{\underset{︸}{\frac{i_{f\beta }\left(t\right)-i_{o\beta }\left(t\right)}{C_{f}}}}$$

From Equation (A5), it can be seen that the derivative path of the voltage can be tracked if the error between the first term and the second (A4) and (A5) is minimized.

## Appendix B

### Secondary Control

Secondary control allows the regulation of voltage and frequency, which are not adjusted by the primary control loop. Implementing a local secondary controller at each distributed generator (DG) results in improved output quality and offers a significant advantage compared to centralized controller approaches [10].

Each DG measures its frequency at each sampling time, averaging the received information from other units and then broadcasting its average version ($\omega_{DG}$) to the other DGs. Then, the consensus data is compared with the nominal frequency of the MG ($\omega_{ref}$) and sent to the secondary controller of $DG_{i}$ to restore the frequency using:

(A6) $$\delta \omega_{DG_{k}}=k{}_{p_{\omega }}\left(\omega_{ref}-\omega_{DG_{k}}\right)+k{}_{i_{\omega }}\int \left(\omega_{ref}-\omega_{DG_{k}}\right)dt$$

(A7) $${\bar{\omega }}_{DG_{k}}=\frac{\sum_{i=1}^{N}\omega_{DG_{i}}}{N}$$

where $k{}_{p_{\omega }}$ and $k{}_{i_{\omega }}$ are the controller parameters of PI and $\delta \omega_{DG_{k}}$ is the compensator signal for primary control (refer to Secondary Control block shown in Figure 6). $\omega_{DG_{k}}$ and $\omega_{ref}$ are the averages of frequency for all DGs and reference frequency of the MG, respectively.

After calculating the average voltage received from the communication network ($\upsilon_{MG}$), the local controller determines the error between this value and the voltage reference $\upsilon_{ref}$ as shown in Equation (A8). Finally, $\delta \upsilon_{DG_{k}}$ is sent to the primary control to compensate for the voltage deviation. The strategy is shown in the Power System layer of Figure 6.

(A8) $$\delta \upsilon_{DG_{k}}=k{}_{p_{v}}\left(\upsilon_{abc}-\upsilon_{DG_{k}}\right)+k{}_{i_{v}}\int \left(\upsilon_{abc}-\upsilon_{DG_{k}}\right)dt$$

(A9) $$\upsilon_{DG_{k}}=\frac{\sum_{i=1}^{N}\upsilon_{DG_{i}}}{N}$$

where $v_{DG_{k}}$ refers to average voltages broadcasted from each DG at the sampling time. Small signal representations of the frequency and voltage for secondary control are detailed in [63].
